# Wheat Plasma Membrane Receptors: Orchestrating Immunity and Bridging to Crop Improvement

**DOI:** 10.3390/cimb48010002

**Published:** 2025-12-19

**Authors:** Hala B. Khalil, Hoda A. Zakherah, Fatimah A. Alhassan, Mai M. Salah, Ahmed M. Kamel, Ammar Y. Mohamed, Haidar A. Alsahoud, Fatma Hamdi Metwaly, Salah A. Mostafa

**Affiliations:** 1 Biological Sciences Department, College of Science, King Faisal University, Al-Ahsa 31982, Saudi Arabia; 2 Biotechnology Program, Faculty of Agriculture, Ain Shams University, 68 Hadayek Shoubra, Cairo 11241, Egypt; 3 Department of Biological Functions Engineering, Graduate School of Life Sciences and Systems Engineering, Kyushu Institute of Technology, Kitakyushu Science and Research Park, 2-4 Hibikino, Kitakyushu 808-0135, Fukuoka, Japan; 4 Department of Biotechnology, Faculty of Agriculture, Cairo University, Giza 12613, Egypt

**Keywords:** wheat, receptors, plasma membrane, signaling cascades, pathogen, biotic stress defense, cross-talk

## Abstract

The plant plasma membrane serves as the primary interface for perceiving extracellular signals, a function largely mediated by plasma membrane receptors (PMRs). In wheat (*Triticum aestivum*), the functional characterization of these receptors is impeded by the species’ large, hexaploid genome, which results in extensive gene duplication and functional redundancy. This review synthesizes current knowledge on wheat PMRs, covering their diversity, classification, and signaling mechanisms, with a particular emphasis on their central role in plant immunity. We highlight the remarkable structural and functional diversification of PMR families, which range in size from 10 members, as seen in the case of wheat leaf rust kinase (WLRK), to over 3424 members in the receptor-like kinase (RLK) family. Furthermore, we reviewed the role of PMRs in being critical for detecting a wide array of biotic stimuli, including pathogen-associated molecular patterns (PAMPs), herbivore-associated molecular patterns (HAMPs), and symbiotic signals. Upon perception, PMRs initiate downstream signaling cascades that orchestrate defense responses, including transcriptional reprogramming, cell wall reinforcement, and metabolic changes. The review also examines the complex cross-talk between immune receptors and other signaling pathways, such as those mediated by brassinosteroid and jasmonic acid receptors, which underpin the delicate balance between growth and defense. Finally, we bridge these fundamental insights to applications in crop improvement, delineating strategies like marker-assisted selection, gene stacking, and receptor engineering to enhance disease resistance. After identifying key obstacles such as genetic redundancy and pleiotropic effects, we propose future research directions that leverage multi-omics, systems biology, and synthetic biology to fully unlock the potential of wheat PMRs for sustainable agriculture.

## 1. Introduction

The plant receptome refers to the full collection of receptors present within the cell, encompassing a diverse range of proteins responsible for sensing and responding to internal and external signals. These receptors are located on the cell surface (plasma membrane), on the outer layers of organelles, or within the cytoplasm, enabling plants to perceive hormones, detect pathogens, and mediate essential growth and immunity processes. This cellular diversity and distribution equip plants with the ability to adapt to varying developmental and environmental cues through a highly specialized and coordinated signaling network [1,2,3]. The signaling networks associated with the plant cell receptome are complex and interconnected, allowing for the integration of multiple signals and the regulation of various cellular processes [4,5,6]. Thus, understanding the components and dynamics of the plant cell receptors is essential for unveiling the molecular mechanisms that underlie these cellular processes and for developing strategies to modulate them for desired outcomes in crop improvement and plant biotechnology.

The comprehensive set of plasma membrane receptors (PMRs) plays essential roles in perceiving signals such as hormones, light, temperature, abiotic stresses, and pathogens [7,8,9,10,11]. Upon signal perception, the receptors initiate a series of molecular events within the cell, leading to signal transduction. This involves the activation or modulation of downstream signaling components, including protein kinases, second messengers, and transcription factors [1,12,13]. PMRs are typically composed of transmembrane proteins [14]. Their influence extends to nearby phospholipid molecules, given their higher hydrophilicity and polarity relative to the hydrophobic phospholipid molecules [15,16]. Notably, transmembrane proteins can pass through the cell membrane [17,18]. Interactions between transmembrane proteins and the lipid bilayer, as well as the water interface, rely on three fundamental properties, namely hydrogen bonding, hydrophobicity, and bipolarity [19,20,21]. These characteristics are essential in regulating the proteins’ interaction with the lipid bilayer and their exposure to the surrounding aqueous environment. Moreover, the lipid bilayer exerts a significant influence on the structural arrangement of membrane proteins, consequently influencing their functionality and activity [22].

**Figure 1:**
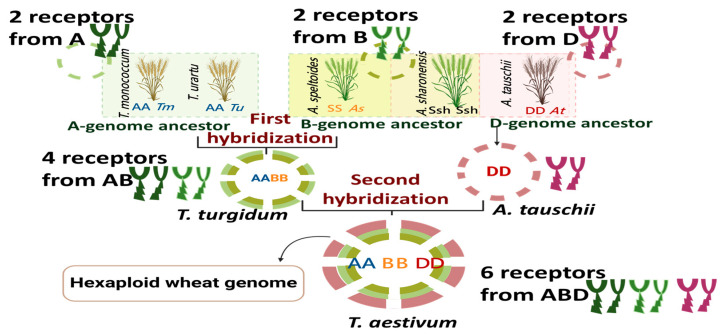
Evolutionary origin of hexaploid bread wheat (*T. aestivum*, AABBDD) leading to an increase in the number of receptors. The A-genome ancestor (*T. urartu*) hybridized with a species related to the B-genome donor (*A. speltoides* or its close relatives such as *A. sharonensis*), giving rise to tetraploid wheat (AABB). A subsequent hybridisation event between tetraploid wheat and the D-genome ancestor (*A. tauschii*) resulted in the formation of hexaploid bread wheat (AABBDD).

The complexity of the wheat genome arises from its status as a hexaploid genome. Bread wheat, *T. aestivum*, possesses a genome consisting of three closely related subgenomes, designated as A, B, and D, which originated from ancestral species [23,24]. These ancestral species underwent hybridization, leading to the formation of hexaploid wheat with a total of 42 chromosomes (2n = 6x = 42). The presence of multiple copies of genes can lead to functional redundancy or divergence, influencing gene expression patterns and protein functions within the wheat plant (Figure 1). The duplication of the genome has also led to challenges in genetic and genomic research in wheat plants. The wheat PMRs represent a complex system that enables plants to detect and respond to a wide array of stimuli, both internal and external.

Wheat PMRs comprise sets of receptors that facilitate the recognition of external signals, including abiotic stressors [25,26], pathogens [27,28,29]. In addition, environmental cues are perceived by photoreceptors, including phytochromes [30], which regulate photoperiodic responses and circadian rhythms, thereby initiating downstream intracellular signaling responses [31,32]. These receptors are integral membrane proteins that are localized on the surface of plant cells, specifically in the plasma membrane. These receptors also play crucial roles in regulating various aspects of wheat plant growth, development, and physiological processes. These networks involve a complex interplay of protein–protein interactions (PPIs), post-translational modifications (PTMs), and signal transduction pathways, leading to the activation or repression of specific genes and the subsequent modulation of plant responses [33,34].

Understanding the wheat PMRs holds immense significance, as it offers the potential to enhance crucial aspects of wheat growth regulation, stress tolerance, and disease resistance. By delving deeper into the PMR-mediated signaling pathways within wheat, we can unravel the molecular mechanisms that govern these processes. This knowledge can pave the way for the development of targeted strategies to manipulate and optimize these pathways, ultimately leading to improved crop performance. A comprehensive study of PMRs and their associated signaling molecules can identify key components that influence agronomically important traits. These traits encompass not only stress tolerance but also disease resistance, nutrient utilization, and yield potential [11,35,36,37]. Manipulating specific PMRs can offer avenues for enhancing disease resistance by activating defense mechanisms against pathogens.

This review provides an overview of the current understanding of wheat PMRs, with a particular emphasis on the role of these receptors in mediating biotic stress responses, including interactions with pathogens, beneficial microbes, and insects. Here, we present recent advances in the identification and functional characterization of wheat PMRs, exploring their signaling networks that govern regulatory mechanisms central to plant immunity and adaptation. Moreover, the review explores the range of methodologies utilized to investigate these receptors, providing critical insights for advancing crop improvement strategies focused on disease resistance and the enhancement of beneficial microbe interactions in wheat. Finally, we address the limitations in current genetic and genomic tools, which often hinder the full understanding of the molecular mechanisms underlying PMR-mediated responses. These challenges underscore the need for more refined techniques and interdisciplinary approaches to effectively investigate PMRs and their potential applications in improving crop resilience.

## 2. Methods for Studying the PMRs

The study of PMRs is central to modern wheat research [38,39]. Due to the complexity of the wheat genome and receptor-mediated signaling, a single approach is inadequate. Instead, a combined use of genomics, transcriptomics, proteomics, and functional biology is necessary to advance from gene identification to understanding function. We now explore these key methods (Table 1).

### 2.1. Genomic and Molecular Techniques

The cornerstone of modern wheat receptor discovery is bioinformatic mining of high-quality reference genomes, such as the IWGSC RefSeq v1.0 and v2.0. These resources have shifted wheat research from a gene-by-gene approach to a systems-level analysis. Researchers can now conduct genome-wide scans for conserved protein domains that are the hallmarks of receptor families. For instance, searches for lysin motif (LysM) domains have identified them as being involved in sensing fungal pathogens [40]. This domain was identified as a part of LysM receptor-like kinases (LysM-RLKs) [41]. Similarly, searches for leucine-rich repeat (LRR) domains have been crucial in identifying numerous potential receptor-like kinases (RLKs), including those involved in sensing bacterial flagellin, such as the flagellin-sensing 2 homologs (FLS2) [42,43]. This approach has also been instrumental in identifying vast families of potential immune receptors, such as cysteine-rich receptor-like protein kinases (CRKs) [44,45]. This in silico prediction provides the essential candidate gene lists that guide all subsequent experimental work.

However, a genomic sequence alone is insufficient. The functional characterization of a receptor mandates the isolation of its full-length coding sequence. This is typically achieved through a combination of PCR-based cloning using degenerate primers designed against conserved domains, followed by rapid amplification of cDNA ends (RACE). This technique has been vital for isolating receptors such as wheat CRK, where obtaining the complete 5′ and 3′ ends was necessary to confirm its domain structure and validate its role in resistance to *Rhizoctonia cerealis* [46].

The progression from identifying a gene sequence to confirming its biological function is driven by sophisticated reverse genetics techniques. Targeting induced local lesions in genomes (TILLING) is a prominent method that combines chemical mutagenesis with high-throughput screening to pinpoint loss-of-function mutations within specific genes. Through TILLING, researchers generate and systematically screen mutant populations to discover alleles that disrupt gene function. This technique has been key in studying plant receptors. For instance, it was used to characterize the Stb6 receptor-kinase-like protein, which controls gene-for-gene resistance to the fungal pathogen *Zymoseptoria tritici* [47].

The recent advent of CRISPR-Cas9 gene editing has revolutionized functional genomics in wheat by providing unparalleled precision. This technology enables the targeted knockout of individual homeologs, allowing researchers to dissect their functional redundancy and additive contributions. A notable example is the TaRPK1, which regulates root architecture, has been modified to improve wheat yield [48]. Using CRISPR-Cas9, researchers generated triple knockouts (across all A, B, and D genomes) to conclusively demonstrate that wheat chitin elicitor receptor kinase 1 (CERK1) is indispensable for chitin signaling and resistance to fungal pathogens. Together, these DNA-based techniques form an integrated toolkit that moves from in silico prediction to functional confirmation, navigating the complexity of the polyploid wheat genome.

### 2.2. Transcriptomic Approaches

Transcriptomic studies have revolutionized the identification of candidate immune receptors in wheat by moving beyond the static genome to capture the dynamic expression profiles of genes during development, stress, and pathogen attack. Early work relied heavily on microarray analysis. For instance, custom-designed arrays have been used to profile gene expression in wheat biotic resistance under *Fusarium graminearum* infection, helping to narrow down RLKs involved in drought response signaling transcripts [49]. The advent of RNA sequencing (RNA-seq) has largely overcome this limitation by providing comprehensive, unbiased transcriptome profiles. RNA-seq enables the detection of alternative splicing, allele-specific expression, and novel genes, thereby significantly refining our understanding of the wheat PMRs [50,51,52]. This has been transformative for wheat receptor discovery. For example, RNA-seq analyses of wheat leaves infected with powdery mildew or rust fungi consistently reveal the concerted upregulation of numerous RLKs, allowing researchers to shortlist key candidates for further functional studies [44,53]. Other complementary transcriptomic methods add further layers of resolution. Quantitative real-time PCR (qRT-PCR) remains the gold standard for validating the expression levels of a small number of candidate receptor genes from RNA-seq or microarray studies due to its superior sensitivity and quantitative accuracy [53,54,55].

Recently, single-cell RNA-seq (scRNA-seq) has begun to be applied to plants, offering the potential to identify which specific cell types express immune receptors, moving from whole-organism resolution to a cellular level, and revealing previously hidden expression patterns [56]. The technique offers a powerful solution to wheat-specific genomic challenges by revealing cell-type–specific expression patterns that bulk RNA-seq cannot resolve due to polyploidy, homoeolog redundancy, and tissue heterogeneity. For example, it has shown that many wheat gene triads display asymmetric homoeolog expression only in specific cell types and identified key regulatory hubs such as SQUAMOSA Promoter Binding Protein-Like 14 (*SPL14*) involved in developmental and signaling pathways [57]. Similarly, single-cell profiling of *Fusarium*-infected tissues uncovered that *F. graminearum* selectively suppresses transcription in vulnerable cell populations, providing mechanistic insight into susceptibility beyond the reach of traditional RNA-seq [58].

### 2.3. Proteomic and PPI Approaches

Proteomic and PPI studies have become indispensable for moving beyond gene-level insights to characterize wheat immune receptors functionally [59]. The complexity of the wheat proteome—dominated by high-abundance storage proteins and further complicated by the hydrophobic nature of membrane-localized receptors—poses substantial analytical challenges. However, advanced mass spectrometry (MS) techniques, particularly liquid chromatography–tandem MS (LC-MS/MS), have enabled large-scale profiling. For instance, phosphoproteomic studies have been pivotal in identifying key signaling events, such as the phosphorylation of the chitin elicitor receptor kinase TaCERK1, a modification essential for its activation and the initiation of immune signaling upon pathogen recognition [60]. Similarly, LC-MS/MS-based analyses have quantified the accumulation of RLKs like wall-associated kinase 1 (WAK1) in response to fungal pathogens, confirming its role in pathogen perception [61].

Complementing proteomic discovery, dedicated PPI mapping techniques are crucial, using approaches such as yeast two-hybrid (Y2H) screens for identifying binary interactions, such as the binding between the cytoplasmic kinase domain of the resistance receptor TaXa21 and specific WRKY transcription factors, a connection that directly links pathogen perception to transcriptional reprogramming [62]. To validate these interactions in a more native cellular context, co-immunoprecipitation coupled with mass spectrometry (Co-IP/MS) is employed. This approach has confirmed the in vivo formation of G-type lectin receptor-like protein kinase (LecRLKs) upon perception of strip rust fungal perception [63]. Furthermore, the bimolecular fluorescence complementation (BiFC) offer visual confirmation and spatial resolution of interactions, as demonstrated by the direct binding observed between the powdery mildew resistance protein Pm55 and its cognate fungal effector [64].

## 3. Structural and Functional Diversity of Wheat PMRs

The diversity of wheat PMRs is fascinating, particularly their remarkable structural variety. The diversity of wheat PMRs is prominently reflected in their structural domains, which determine each receptor’s functionality and specificity for various extracellular signals. Equipped with a wide repertoire of domains and motifs, these receptors perform distinct cellular roles, enabling interactions with a broad spectrum of ligands [1]. This allows them to mediate specific cellular responses, helping wheat plants adapt and thrive in dynamic environmental conditions [65]. Consequently, PMRs serve as the interface between the external environment and the internal cellular responses, enabling plants to perceive and integrate various signals [11,35,36,37,54]. Ultimately, the rich diversity of wheat receptors reflects the complex and dynamic nature of the plant’s interactions with its surroundings.

### 3.1. G Protein-Coupled Receptors (GPCRs)

**Figure 2:**
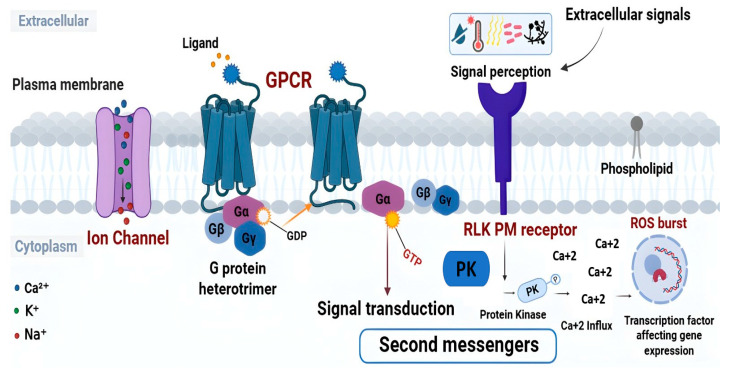
Schematic overview of plasma membrane–mediated signal perception and transduction in plant immunity. Components of plasma membrane signaling pathways involved in perceiving extracellular cues. Ion channels regulate the movement of Ca^2+^, K^+^, and Na^+^ across the membrane, GPCRs, heterotrimeric G-proteins, and PM-RLKs relay signals upon ligand perception and activate downstream second messengers.

G protein-coupled receptors (GPCRs) are crucial cell surface receptors in plants. These receptors have a conserved structure with seven transmembrane domains, connected by intracellular and extracellular loops, which enables their interactions with diverse ligands [66,67]. These receptors, characterized by their transmembrane topology and involvement in diverse signaling pathways, play a fundamental role in translating extracellular signals into intracellular responses, ultimately influencing growth, development, and stress adaptation in wheat. The unique architecture of GPCRs includes a transmembrane domain responsible for ligand binding and activation, coupled with intracellular domains that interact with G proteins, mediating downstream signal transduction. In wheat, GPCRs enhance the plant’s capacity to tolerate and adapt to challenging conditions (Figure 2). These receptors perceive stress-induced signaling molecules and activate defense mechanisms. Furthermore, GPCRs in wheat are implicated in the regulation of immune responses [68]. By recognizing pathogen-derived molecules, such as chitin or peptide elicitors, GPCRs activate defense signaling pathways, resulting in the production of antimicrobial compounds and the induction of defense-related genes. This dual role in perceiving stress signals and pathogens underscores the pivotal function of GPCRs in shaping wheat’s strategies for adaptation and survival.

### 3.2. Ion Channel Receptors

Ion channel receptors in wheat constitute a vital component of the plant’s intricate signaling network, governing the flow of ions across cellular membranes and playing a fundamental role in diverse physiological processes. These receptors, characterized by their ion channel domains, facilitate the movement of ions such as calcium (Ca^2+^), potassium (K^+^), and sodium (Na^+^) across cell membranes, enabling wheat plants to swiftly respond to various environmental cues and regulate cellular responses accordingly [29]. The distinctive structure of ion channel receptors features transmembrane domains that form pores through which ions selectively pass. In wheat, these receptors are involved in modulating cellular processes like osmotic regulation, nutrient uptake, and signal transduction (Figure 2). For instance, the wheat ion channel receptor TaTPC1 responds to salt stress by regulating Na^+^ and K^+^ homeostasis. Through this mechanism, TaTPC1 aids in the maintenance of ion balance and cellular turgor, ultimately influencing the plant’s ability to withstand osmotic stress [69]. Moreover, ion channel receptors play a key role in wheat’s response to biotic stress. During pathogen attack, these receptors regulate ion fluxes across the plasma membrane, activating downstream defense signaling. Ion channel activation alters membrane potential and triggers the release of secondary messengers such as Ca^2+^, initiating signaling cascades that ultimately lead to the expression of defense-related genes [70].

### 3.3. Receptor-like Protein Kinases

Wheat receptor-like protein kinases (RLKs) represent a diverse and essential group of transmembrane receptors that play a central role in cellular communication and coordination within plants (Figure 2). As key components of the cell surface, RLKs are involved in perceiving extracellular signals and transducing them into intracellular responses, enabling plants to adapt to their ever-changing environments [71,72]. Specifically, these receptors are critical for biological processes such as growth, defense, and stress adaptation [45,72,73]. The hexaploid genome of bread wheat has facilitated a remarkable expansion and diversification of RLK families, resulting in a sophisticated and redundant signaling network. This genomic abundance presents opportunities for resilience, particularly in terms of the balance between functional redundancy among paralogs and the evolution of novel, pathogen-specific recognition capacities [24]. Comparisons with Arabidopsis show that wheat maintains core immune modules but also features lineage-specific adaptations, including expanded receptor families and unique resistance genes from wild relatives.

**Figure 3:**
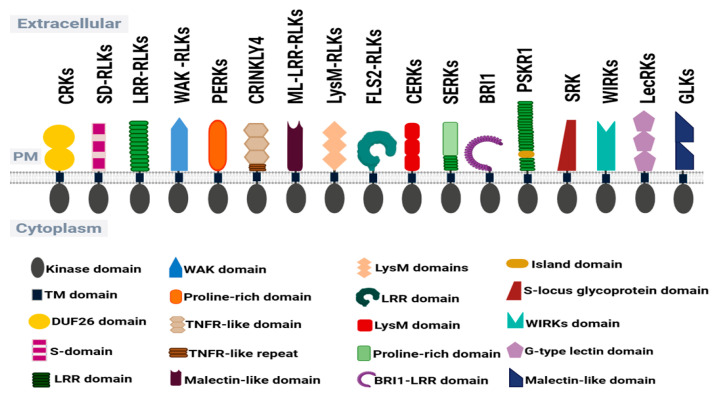
Schematic representation of major receptor-like kinases and their characteristic extracellular domains in wheat plants.

Research has revealed their considerable structural diversity, ligand specificity, and involvement in intricate signaling networks, making them central to understanding the complexities of plant signaling pathways [45,74,75]. The variety of domains associated with these kinases reflects the wide range of signaling processes plants must manage in response to both internal and external cues (Figure 3; Table 2). Wheat RLKs are particularly important because some confer resistance to major fungal diseases—such as rusts, powdery mildew, and Fusarium head blight—while others regulate key agronomic traits including grain size, plant architecture, and tolerance to abiotic stresses. The extracellular domains of RLKs contain various motifs that enable them to recognize a broad array of ligands, including hormones, pathogen-associated molecular patterns (PAMPs), and other environmental cues [72,74,76]. The diversity of the extracellular domains defines their functional specialization. Ligand binding triggers conformational changes in the intracellular kinase domains of RLKs, leading to autophosphorylation and the initiation of downstream signaling cascades [71,77,78]. Below is a list of the major RLK families in wheat, summarizing their functions as detailed in Table 2.

#### 3.3.1. RLKs as Core Pattern Recognition Receptors

Families like the cysteine-rich RLKs (CRKs), S-domain RLKs (SD-RLKs), and leucine-rich repeat RLKs (LRR-RLKs) serve as primary sentinels. CRKs, with their DUF26 motifs, are significantly expanded in wheat and are implicated in ROS signaling and responses to both biotic and abiotic stresses [79,80,81,82,83,84,85,86,87]. Their proliferation suggests a mechanism for robust signal amplification, though whether individual paralogs have acquired distinct ligand specificities remains debated. SD-RLKs, often containing lectin motifs, include well-characterized examples such as LRK10L, which confers powdery mildew resistance [88,89]. Comparatively, while chitin perception is a conserved function, the extent to which wheat SD-RLKs recognize other wheat-specific PAMPs is unclear. The LRR-RLK family is the largest and most versatile, encompassing legendary broad-spectrum resistance genes like *Lr34*/*Yr18*/*Pm38* [90] and specific receptors for Fusarium head blight (TaTLRK-6D) [27] and stripe rust (*Yr5*, *Yr7*) [91]. The expansion of LRR-RLKs in wheat likely enhances pathogen recognition capacity, but how these receptors balance their overlapping roles in immunity and development, such as the brassinosteroid signaling mediated by BRI1, remains under extensive study [92,93,94].

#### 3.3.2. RLKs as Sensors of Cell Wall Integrity and Damage

The wheat cell wall is a critical barrier, and several RLK families are dedicated to monitoring its status. Wall-associated kinases (WAKs) and proline-rich/extensin-like RLKs (PERKs) are central to this function. WAKs, such as TaWAK6, directly bind pectin and sense damage during pathogen attack (e.g., leaf rust), linking mechanical integrity to immune activation [95,96,97,98,99]. PERKs represent a large wheat-specific family (~30–37 genes) with stress-responsive expression, suggesting a specialized role in cell wall signaling during drought and pathogen assault [100,101]. An unresolved question for both families is the precise identity of their physiologically relevant ligands during different stress conditions.

#### 3.3.3. RLKs as Specialized Recognizers of Microbial Signatures

Certain RLKs have evolved exquisite specificity for conserved microbial molecules. The lysin motif RLKs (LysM-RLKs) and chitin elicitor RLKs (CERKs) families are essential for perceiving fungal chitin. In wheat, TaCEBiP and TaCERK1 interact to activate the defense against pathogens such as *F. graminearum* and *Zymoseptoria tritici* [102,103,104]. Comparative studies show the core chitin signaling pathway is conserved, but the functional coordination of multiple homoeologs in wheat’s polyploid genome is complex and not fully understood. Similarly, lectin RLKs (LecRLKs) with diverse carbohydrate-binding domains (L-, G-, C-types) perceive fungal PAMPs like chitin and β-glucans. Examples include LecRK-V and TaSRLK, which enhance resistance to powdery mildew and stripe rust, respectively [63,105]. The flagellin-sensing 2 (FLS2) receptor provides conserved recognition of bacterial flagellin [106], though its ecological significance in wheat disease scenarios warrants further study.

#### 3.3.4. RLKs as Regulators of Growth and Defense

Wheat RLKs are pivotal integrators of growth and stress responses. The brassinosteroid-insensitive 1 (BRI1) receptor governs fundamental growth processes, and its manipulation has been central to improving wheat architecture [107,108,109]. Phytosulfokine receptors (PSKRs) integrate peptide hormone signals to promote cell proliferation while modulating stress responses [110,111,112]. Somatic embryogenesis receptor kinases (SERKs) function as indispensable co-receptors for multiple RLKs, including BRI1 and immune receptors, amplifying signals for both development and defense [113,114,115]. CRINKLY4 (CR4), though less studied in wheat, is predicted from ortholog function to regulate epidermal patterning and grain development [116,117,118]. A major unresolved debate across these families involves understanding the precise molecular mechanisms that prioritize and allocate resources between growth and defense programs under varying environmental pressures.

#### 3.3.5. Non-Canonical RLKs

Some wheat RLKs exhibit unique structural or functional adaptations. Malectin-like LRR-RLKs (ML-LRR-RLKs), such as RLK-V from *Haynaldia villosa*, combine glycan-binding malectin domains with LRRs to confer powdery mildew resistance [119]. S-locus RLKs (SRKs) in wheat have been neofunctionalized from self-incompatibility proteins and are often found as tandem kinases that collaborate with NLR receptors for race-specific rust resistance [120]. Wound-induced RLKs (WIRKs) such as TtdLRK10L-1 act as rapid-response sentinels, coupling damage perception to localized defense activation [89,121]. Finally, GHR1-like Kinases (GLKs) represent a fascinating divergence, possessing a pseudokinase domain that functions as a signaling scaffold. TaGLKs are crucial for integrating ABA and ROS signals to orchestrate stomatal closure, directly linking RLK signaling to drought tolerance [46,122].

Despite their structural differences, GPCRs, ion channels, and receptor-like kinases (RLKs) form an integrated immune network in wheat. Although plants lack canonical GPCRs, G-proteins function downstream of multiple PRRs as integrators of immune signals [123]. G-protein components can functionally associate with RLKs to fine-tune kinase activity and dynamically balance growth and defense. Moreover, full NLR-mediated resistance depends on intact RLK/RLP signaling, as disruption of coreceptors or RLCKs severely compromises NLR outputs [124]. This convergence is coordinated through shared cytoplasmic kinases and secondary messengers. Following ligand perception, LRR- and S-domain RLKs typically heterodimerize with SERK/BAK1 coreceptors [125]. The activated complexes phosphorylate receptor-like cytoplasmic kinases, particularly BIK1 and RLCK-VII members, which function as central signaling hubs. These RLCKs directly phosphorylate plasma membrane ion channels to trigger Ca^2+^ influx and activate the NADPH oxidase, generating the rapid oxidative burst [124].

## 4. Expansion of Wheat PMR Families

**Figure 4:**
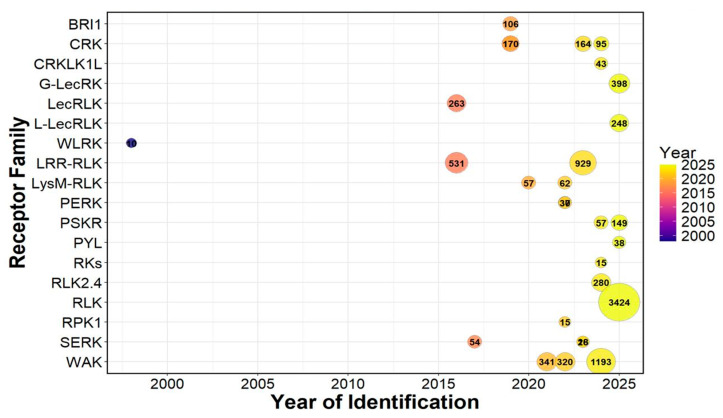
Diversity of RLK families. The number of identified members for major RLK families in wheat is shown, as reported in various studies. The numbers in the circles indicate the abundance of members belonging to each gene family.

Over the past three decades, substantial efforts have been dedicated to identifying and characterizing wheat PMRs. The first family to be identified was the wheat leaf rust kinase family, WLRK, described by Feuillet et al. [73]. This family consisted of 10 members that were assigned to specific chromosomes and conferred disease resistance. Subsequent studies, enabled by the high-quality IWGSC wheat genome sequence, have achieved a more comprehensive identification and characterization of the PMR families. The extensive expansion observed across PMR families is strongly influenced by wheat’s allohexaploid genome structure, which promotes the retention of homeologous RLKs across the A, B, and D subgenomes and enables subfunctionalization, thereby increasing receptor diversification and flexibility in immune responses [126]. Since then, numerous RLK families have been reported, with member numbers varying across studies due to improvements in genome assembly, annotation pipelines, and experimental objectives (Figure 4). Among these, the LRR-RLK family represents one of the largest, with 531 members initially identified [127] and later expanded to 929 members in a more recent study [42]. Similarly, a global analysis of the general RLK gene repertoire identified more than 3424 RLK genes in wheat [126], highlighting the extraordinary expansion of this gene family in polyploid cereals. This large-scale expansion aligns with widespread gene duplication and retention events that have enhanced the structural and functional diversity of receptor families across the wheat genome.

Other families have been characterized by more moderate gene numbers. The BRI1 gene family comprises 106 members [128], while the CRKs have been reported to variable numbers depending on study design: 170 genes [129], 164 genes during leaf rust infection [44], and 95 genes under high-temperature stress [43]. The *Catharanthus roseus* RLK1-like family (CrRLK1L) has 43 members [130], while lectin-type RLKs also show strong expansion: 263 general LecRLKs [131], 248 L-type LecRLKs [126], and 398 G-type LecRLKs [63]. Smaller RLK families have also been identified with specialized functions. The LysM-RLKs, known for their role in chitin recognition, include 57–62 members [132,133]. The PERK family contains between 30 and 37 members [100,101]. PSKRs have been identified with 57 members [111] and later expanded to 149 members [134]. The pyrabactin resistance 1-like (PYL) receptors number 38 members [135], while the RPK1 family comprises 15 members [45]. Other gene families have been functionally associated with embryogenesis and immunity. The SERKs range from 18 to 54 members depending on the study [114,136,137]. The TaRLK2.4 subgroup was reported with 280 members [138]. The WAKs, key players in pathogen resistance, display remarkable expansion, ranging from 320–341 members [139,140] to as high as 1193 members in a recent phylogenetic analysis [141].

This expansion creates a framework where functional redundancy and neofunctionalization arise from gene duplications and subgenomic divergence, allowing for the emergence of novel immune functions within specific wheat lineages [126]. In addition, the massive expansion of the receptor gene repertoire in wheat creates a sophisticated, multi-layered regulatory system for disease resistance. Rather than relying on a few key genes, wheat possesses a network of homeologous and paralogous receptors that can be differentially regulated [126]. This allows for precise, tissue-specific, and temporally controlled immune responses. Different receptor paralogs can be selectively activated in response to distinct pathogens or developmental cues, ensuring a targeted defense without unnecessary metabolic cost [139,140]. Furthermore, subfunctionalization among expanded family members enables the partitioning of roles, where some receptors may specialize in recognizing conserved microbial patterns while others fine-tune sensitivity or modulate downstream signaling cascades. This regulatory complexity means that while some receptor variants may confer broad-spectrum basal resistance, others can be fine-tuned through expression or allelic variation to provide highly specific, race-specific resistance.

## 5. Wheat PMRs’ in Response to Biotic Challenge

Wheat plants have evolved sophisticated mechanisms to recognize and respond to various biotic challenges presented by pathogens, herbivores, and symbiotic organisms. At the forefront of these interactions are PMRs, which play a pivotal role in perceiving and transducing biotic signals to initiate appropriate cellular responses [62,142]. The participation of plant PMRs in biotic interactions is a dynamic and complex process that involves the recognition of specific molecular patterns and subsequent activation of signaling pathways [11,143]. These receptors possess extracellular domains responsible for ligand recognition and intracellular domains involved in downstream signal transduction. They have the ability to recognize a wide range of biotic molecules, including PAMPs, herbivore-associated molecular patterns (HAMPs), and symbiotic signals [11,36,37,144,145]. These cascades often involve the recruitment and activation of other signaling components, such as kinases, phosphatases, and transcription factors, which ultimately regulate gene expression and cellular responses [37,146,147,148,149]. The ability of wheat PMRs to recognize and respond to a wide range of biotic molecules highlights their crucial role in mediating plant interactions with various organisms. Through their selective ligand-binding capacity and downstream signal transduction, these receptors enable plants to mount appropriate defense responses against pathogens, deter herbivory, or establish symbiotic relationships [11,37,144,145]. The diversity of PMRs and their ligand specificities provides the plant with a sophisticated molecular toolkit to respond effectively to the complex and ever-changing biotic challenges it faces.

### 5.1. Wheat Defense Against Pathogens

Wheat plants employ a multilayered defense system to counteract a wide array of pathogens. These defense layers involve various mechanisms that act at different stages of pathogen attack [150,151]. The participation of PMRs is integral to this system, as they play a crucial role in perceiving pathogenic cues and initiating appropriate defense responses. PMRs participate in these defenses by triggering a series of signaling events that prepare the plant for subsequent defense responses [103,148].

#### 5.1.1. Involvement of Wheat PMRs in Pattern-Triggered Immunity (PTI)

**Figure 5:**
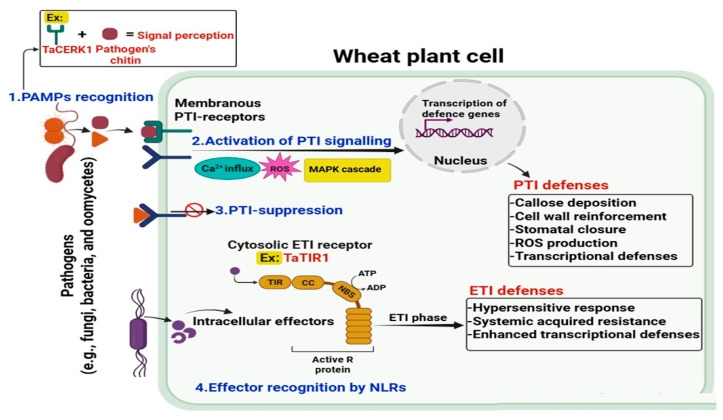
Overview of PTI and ETI defense mechanisms in wheat plant cells. The diagram illustrates the two-tiered immune system of wheat in response to pathogen attack. First, PAMPs are recognized by PMRs like TaCERK1 on the plasma membrane, initiating PTI. Second, PTI activation involves Ca^2+^ influx, ROS production, and MAPK cascade signaling, leading to transcriptional activation of defense genes and PTI defenses, including callose deposition, cell wall reinforcement, stomatal closure, ROS accumulation, and transcriptional defenses. Third, some pathogens deliver effectors to suppress PTI responses.

PMRs are vital components of pattern-triggered immunity (PTI), as they directly perceive PAMPs and mediate the subsequent defense responses (Figure 5). Recognition of PAMPs by wheat PMRs is essential for initiating plant defense responses against pathogens known as PRRs [27,36,84,152,153,154,155]. Activation of PRRs triggers a cascade of key signaling modules, encompassing rapid phosphorylation of receptor-like cytoplasmic kinases (RLCKs), influx of Ca^2+^ across the plasma membrane, burst of ROS, and activation of CDPKs, MAPK cascades, and heterotrimeric G-protein [37,156,157,158]. One well-studied example of PAMP recognition in wheat is the perception of bacterial flagellin, a key PAMP derived from flagellar proteins [159,160,161]. The flagellin-sensing pathway in wheat involves the recognition of a receptor-like kinase called wheat FLS2 that can recognize flagellin-derived peptides, such as flg22, and initiate downstream signaling events leading to the activation of defense responses [162]. Generally, in plants, when flg22 is recognized by FLS2, it associates with the co-receptor BRI1-associated kinase 1 (BAK1), resulting in the activation of downstream signaling pathways that lead to the production of antimicrobial compounds, reinforcement of cell walls, and induction of defense-related genes [78]. Another example of PAMP recognition in wheat involves the perception of chitin, a major component of fungal cell walls [37]. Chitin is recognized by wheat CERKs. For instance, Wheat Chitin Elicitor Receptor Kinase 1 (TaCERK1) has been shown to recognize chitin and initiate defense responses in wheat [163].

Other PAMPs recognized by wheat receptors include fungal cell wall-derived molecules and bacterial lipopolysaccharides. For instance, wheat RLKs have been identified as putative receptors involved in the recognition of fungal cell wall components, such as fungal chitin [164]. These RLKs play a role in activating defense responses against fungal pathogens. Furthermore, PRRs have been implicated in the recognition of bacterial lipopolysaccharides, which are characteristic components of Gram-negative bacteria. The perception of lipopolysaccharides by plant receptors triggers immune signaling pathways and activates defense responses [165]. Moreover, Wang et al. [166] demonstrated that wheat perceives phytocytokines through cell surface receptor kinases for initiating immune signaling pathways. This mediates wheat resistance to *Xanthomonas translucens*, *F. graminearum*, and *F. pseudograminearum*. The studies of PAMP recognition by wheat receptors have greatly advanced our understanding of plant immunity and the molecular basis of pathogen recognition in crops. These discoveries provide valuable insights into the mechanisms of plant-pathogen interactions and offer opportunities for developing novel strategies to enhance plant resistance against pathogens. Further research on wheat receptors involved in PAMP recognition, their ligand specificities, and downstream signaling pathways will continue to shed light on the intricate molecular mechanisms underlying plant defense responses.

The activation of the Toll/interleukin-1 receptor (TIR) signaling by PMRs in plants plays a crucial role in initiating immune responses against pathogens. TIR signaling is a conserved pathway involved in recognizing PAMPs and activating defense mechanisms. In wheat, several PMRs have been identified to activate TIR signaling upon pathogen perception (Figure 5). One example is the TaTIR1 (TIR-NBS-LRR 1) receptor in wheat, which contains a TIR domain [167]. Another example is the TaTIR-NB-LRR class of receptors, which possess both TIR and nucleotide-binding (NB) domains [168]. These receptors are involved in recognizing specific effectors and activating TIR signaling in wheat. For instance, TaTIR-NB-LRR genes, such as Resistance to Cereal Rusts 1 (TaRCR1) and TaLr10, confer resistance against rust pathogens by activating TIR-mediated immune responses [169]. These components regulate the expression of defense genes, the production of antimicrobial compounds, and the reinforcement of cell wall integrity, collectively enhancing the plant’s defense against pathogens.

#### 5.1.2. Involvement of Wheat PMRs in Effector-Triggered Immunity (ETI)

ETI in wheat is a critical defense mechanism that involves the participation of PMRs. ETI occurs when specific pathogen effectors, which are virulence factors secreted by pathogens, are recognized by plant receptors encoded by resistance (R) genes [170]. This recognition activates a signaling cascade that leads to the activation of defense responses, including the HR and the induction of defense-related genes [171,172]. Several PMRs have been identified to participate in ETI, highlighting their crucial role in pathogen recognition and defense activation (Figure 5). One example of ETI in wheat is the recognition of the AvrPm3 effector by the *Pm3* gene, which encodes nucleotide-binding (NB-ARC) and LRR domain proteins [173,174,175]. *Pm3* is an R gene in wheat that confers resistance against the fungal pathogen Bgt, which causes powdery mildew disease [174]. The AvrPm3 effector is specifically recognized by the Pm3 PMR, leading to the activation of ETI-associated responses, including HR and resistance against powdery mildew. This interaction between the AvrPm3 effector and the Pm3 receptor has been studied extensively, providing insights into the mechanisms of effector recognition and the downstream signaling events that mediate defense responses. Further, powdery mildew effectors, including AVRPM3 variants and SVRPM3a1/f1, form homo- and heteromeric complexes that determine allele-specific, *Pm3*-mediated resistance, with subtle structural variations dictating recognition specificity [176]. Another example involves the recognition of the AvrSr50 effector by the Sr50 PMR in wheat. *Sr50* is an R gene that confers resistance against the stem rust fungus (*P. graminis* f. sp. *tritici*). The AvrSr50 effector is specifically recognized by the Sr50 receptor, resulting in the induction of ETI and subsequent resistance against stem rust [177]. In addition, the AvrLr10 and AvrL21 effectors are specifically recognized by the *Lr10* and Lr21 PMRs, leading to the activation of ETI and enhanced resistance to leaf rust [178,179,180]. *Rpg1* is an R gene in wheat that confers resistance against the stem rust fungus *P. graminis*. The AvrRpg1 effector is recognized by the Rpg1 PMR, leading to the activation of ETI and resulting in enhanced resistance to stem rust [181]. *Yr10* is an R gene in wheat that confers resistance against the stripe rust fungus *P. striiformis*. The AvrYr10 effector is specifically recognized by the Yr10 PMR, initiating ETI and resulting in resistance to stripe rust [182]. Another study shows that *Zymoseptoria tritici* secretes effectors that suppress programmed cell death. Despite low sequence similarity, many effectors share conserved folds that interact with receptor signaling components, subverting immune activation. By modulating these pathways, the effectors promote fungal colonization during the latent phase, demonstrating how pathogens manipulate host signaling to evade immunity [183]. These examples of ETI in wheat highlight the pivotal role of PMRs in perceiving specific pathogen effectors and initiating defense responses. The recognition of effectors by PMRs triggers a complex network of signaling events, leading to the activation of defense-related genes, the production of antimicrobial compounds, and the HR, ultimately enhancing the plant’s resistance against the corresponding pathogens. Understanding the molecular mechanisms underlying ETI and the involvement of PMRs in wheat is essential for developing strategies to enhance plant resistance and mitigate the impact of pathogen infections.

### 5.2. Wheat PMRs as Guardians of Insect Resistance

Plants have evolved a sophisticated defense system to counteract the threats posed by herbivorous insects. Central to this defense system are the PMRs that recognize specific HAMPs [144,184]. The recognition of HAMPs by wheat PMRs is a fundamental step in initiating the plant’s defense responses, enabling it to mount an effective defense against insect pests [185]. Upon the recognition of HAMPs by wheat PMRs, a cascade of defense responses is activated. This recognition triggers intracellular signaling events, leading to the modulation of gene expression and the production of defense-related compounds [186].

The specificity of the PMRs in recognizing HAMPs allows wheat plants to fine-tune their defense responses. Different receptors may have distinct affinities for specific HAMPs, enabling the plant to respond differentially to various herbivores or different stages of herbivore attack. This specificity also contributes to the adaptation and co-evolutionary dynamics between wheat plants and their herbivorous counterparts, as receptors and HAMPs continuously interact and evolve to gain an advantage in the ongoing arms race between plants and insects. For instance, RLKs are a prominent class of PMRs involved in wheat’s defense against insect pests [187,188]. The activation of defense responses through PMRs and RLKs in wheat confers enhanced resistance against herbivory. By detecting herbivore-associated molecular patterns (HAMPs) and activating defense signaling pathways, wheat plants can effectively limit damage caused by herbivorous insects [187]. Moreover, wheat plants can also release volatile organic compounds (VOCs) as part of their defense mechanisms. These VOCs attract natural enemies of herbivores, such as parasitoids or predators, which can further aid in pest control [189,190].

### 5.3. Wheat PMRs in Symbiotic Microbial Interactions

PMRs play a critical role in mediating symbiotic interactions with beneficial microorganisms, including arbuscular mycorrhizal (AM) fungi and plant growth-promoting rhizobacteria [191]. These receptors, primarily RLKs and receptor-like proteins (RLPs), enable wheat to recognize symbiotic signals and initiate intracellular signaling that facilitates mutualistic associations. For example, TaWAK-6D has been shown to modulate defense gene expression in wheat during fungal interactions while potentially playing roles in symbiotic signaling [28]. Similarly, LysM domain receptor-like kinases, essential in legumes for Nod factor recognition in rhizobial symbiosis, have participated in AM fungal recognition and conferred symbiosis [192]. These receptors facilitate membrane trafficking events required for symbiotic interface formation, such as the periarbuscular membrane in AM symbiosis, which involves extensive remodeling of the host plasma membrane. Components of the SNARE complex, involved in vesicle fusion, also play roles in receptor trafficking and symbiosome formation in related systems, underscoring the complexity of receptor-mediated signaling in wheat symbiotic interactions [193]. These receptor-mediated pathways optimize nutrient exchange, enhance stress resilience, and improve overall wheat productivity through enhanced symbiotic capacities.

## 6. Receptor Cross-Talk Across Various Signaling Pathways

The foundation of signal perception in wheat lies in the specific molecular interaction between a ligand and its receptor, a process governed by the structure of ligand-binding domains (LBDs). These domains are critical components that determine the recognition and specificity of ligands, initiating precise cellular responses that govern plant physiology and development [1,65]. The remarkable diversity of LBDs within the wheat PMRs enables the perception of a vast array of signals, including hormones, signaling peptides, nutrients, and PAMPs. However, signaling pathways do not operate in isolation. The wheat receptors function as an integrated network characterized by extensive receptor cross-talk, which refers to the intricate communication and interaction between distinct signaling pathways. This cross-talk allows the plant to process multiple simultaneous signals and prioritize responses to dynamically changing conditions.

**Figure 6:**
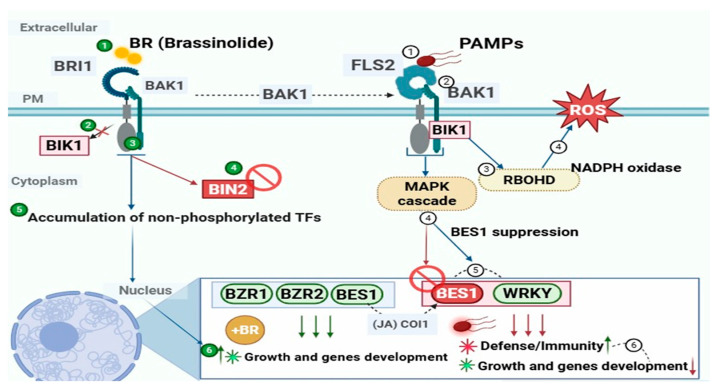
Crosstalk between brassinosteroid signaling and PTI pathways in plants. Brassinolide binds to its receptor BRI1, which forms a complex with BAK1, leading to the activation of downstream signaling. This interaction inhibits BIN2, promoting the accumulation of non-phosphorylated transcription factors (TFs) such as BZR1, BZR2, and BES1, which regulate genes involved in plant growth and development.

A prime example of sophisticated receptor signaling in plants is the perception of BR hormones by the wheat BRI1 receptor (Figure 6). In wheat, BRI1 possesses an extracellular LRR domain that acts as the binding site for BR [194]. This binding triggers a defined signaling cascade: TaBRI1 dissociates from its inhibitory protein BKI1, undergoes autophosphorylation, and associates with the co-receptor BAK1 [185,194]. Downstream, the brassinosteroid-insensitive 2 (BIN2) acts as a critical negative regulator. In the absence of BR, BIN2 phosphorylates the transcription factors BZR1 and BZR2/BES1, leading to their cytoplasmic retention or degradation. Upon BR perception, BIN2 is inhibited, allowing dephosphorylated BES1/BZR1 to accumulate in the nucleus and regulate BR-responsive genes thereby coordinating growth and development [195,196].

This pathway does not operate in isolation, and a key illustration of hormonal cross-talk is its interaction with the jasmonic acid (JA) pathway. While BR signaling promotes growth, JA signaling, mediated by its specific receptors such as COI1, activates defense responses [194,197]. This cross-talk allows wheat to balance resource allocation between growth and defense [197]. Furthermore, cross-talk occurs through direct collaboration between different receptor classes during resistance. For instance, FLS2 and RLP EF-Tu Receptor (EFR) form complexes to recognize bacterial pathogens [198]. Activation of FLS2 triggers cytoplasmic kinases, including BIK1, and a MAPK cascade, enabling the activation of NADPH oxidase and ROS production. This immune signaling actively suppresses the BR-activated transcription factor BES1 in the nucleus, shifting gene expression from growth to defense, a process mediated by WRKY transcription factors [27,196,199]. JA signaling via the COI1 receptor reinforces this suppression of BES1, further bolstering WRKY-mediated immunity and antagonizing BR signaling [200,201].

Another critical layer of this signaling network involves abscisic acid (ABA), which mediates the trade-off between abiotic stress adaptation and biotic defense. In wheat, ABA perception is mediated by the PYR/PYL/RCAR receptor family, including plasma membrane-associated members such as TaPYL9 and TaPYL4-like proteins [202,203]. Upon ABA binding, these receptors inhibit clade A PP2C phosphatases, thereby activating SnRK2 kinases that phosphorylate downstream transcription factors to restrict growth and promote stomatal closure [204]. The ABA pathway also interacts directly with plasma membrane immune receptors; for example, the FLS2/BAK1 complex is modulated by ABA-responsive kinases and phosphatases that can attenuate immune signaling to favor abiotic stress resilience [205]. Salicylic acid (SA) and JA pathways further intertwine with these networks, as SA-regulated components such as NPR1 and WRKY70 influence JA/ethylene modules, with SA often antagonizing JA-dependent defenses to fine-tune basal and inducible immunity while interacting with BR-responsive BES1/BZR1 growth regulators. In wheat, SA- and JA-associated WRKY transcription factors converge with BR and ABA signaling nodes on shared downstream gene networks, providing a mechanistic framework for multi-hormone integration of pattern-triggered immunity and receptor-mediated growth regulation at PMR-centered transcriptional hubs [206]. Collectively, this coordinated cross-talk among receptor pathways produces a more robust and adaptable defense architecture than any pathway operating independently.

## 7. Exploiting the Wheat Receptors for Crop Improvement

**Figure 7:**
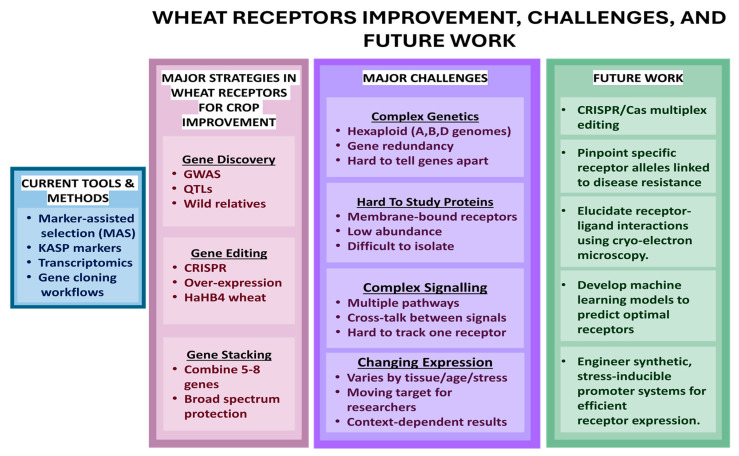
Overview of strategies, challenges, and future directions in wheat receptor research for crop improvement. The diagram summarizes the current tools and methodologies used in wheat receptor studies.

Strategies to exploit PMRs for wheat improvement focus on leveraging their central role in sensing pathogen cues and activating intracellular signaling pathways that regulate growth, development, and stress responses. By identifying key receptor proteins associated with resistance to pathogens, researchers aim to manipulate these receptors through genetic, genomic, and molecular breeding approaches. Techniques such as marker-assisted selection (MAS) facilitate the introgression of receptor genes linked to desirable traits (Figure 7), especially by utilizing high-throughput genotyping platforms to track specific receptor allele introgression from wild relatives [207]. For instance, high-resolution mapping has enabled the precise transfer of resistance loci from *A. tauschii* into elite wheat cultivars while minimizing linkage drag, thereby broadening the genetic base of receptor-mediated immunity. Furthermore, genome editing tools, including CRISPR, enable precise modifications to enhance receptor function (Figure 7). Beyond simple gene knockouts, next-generation editing technologies, such as base editing and prime editing, now offer the potential to engineer precise amino acid changes within the LBDs of PMRs [208]. This precision allows for the creation of novel receptor variants with expanded recognition specificities for evolving pathogen effectors without introducing foreign DNA [209]. The integration of advanced tools like transcriptomics, CRISPR/Cas9, and kompetitive allele specific PCR (KASP) markers further clarifies receptor functions within host–pathogen interactions, enabling precise gene validation and accelerating the development of improved wheat varieties [47,210,211,212]. This progress is fueled by streamlined gene cloning workflows that efficiently identify and characterize receptors, such as the disease resistance gene *Sr6* [195]. By reducing the time and space needed for mutant screening, these protocols rapidly pinpoint critical receptor alleles and shorten the timeline for cloning resistance genes from years to months, enabling the rapid deployment of functional receptor haplotypes. Complementing these rapid cloning methods, the modification of wheat receptors continues to be revolutionized by genetic engineering. By overexpressing critical receptors like RLK-PMRs, scientists can confer robust resistance and abiotic stress tolerance. The success of the *HaHB4* transgene wheat demonstrates this potential. For instance, the world’s first commercial drought-tolerant GM wheat, HB4 wheat, was developed and released in Argentina in 2020. This transgenic wheat carries the *HaHB4* gene from sunflower, which activates the drought stress response in wheat, alongside a *BAR* gene from *Streptomyces hygroscopicus* that provides resistance to the herbicide glufosinate [209,213]. Furthermore, the frontier of receptor engineering now includes domain-swapping and the design of synthetic receptors. Recent advances in receptor biology have enabled wheat-focused domain-swapping strategies based on endogenous immune receptors. For example, several wheat LRR-RLKs such as TaFLS2, TaCERK1, and TaRLK1 show natural variation in their extracellular domains that affects pathogen recognition. By experimentally swapping ectodomains from high-responsiveness alleles with intracellular kinase domains of strongly signaling wheat RLKs, researchers can generate chimeric receptors optimized for both recognition and signaling within the wheat cellular environment. Such wheat-specific chimeras build directly on natural receptor variants already present in *T. aestivum* and its progenitors, providing a biologically grounded path for engineered recognition without relying on noncereal receptors [207,210].

Stacking multiple receptor genes offers durable, broad-spectrum resistance for wheat improvement. This approach deploys multiple defense pathways simultaneously, reducing the risk of resistance breakdown. Gene stacking involves combining several resistance genes into a single locus or cassette, allowing simultaneous expression of multiple defense mechanisms against pathogens and environmental stresses. This strategy, known as pyramiding, is most effective when stacking receptors with distinct functions. This multi-layered defense forces pathogens to overcome multiple recognition barriers simultaneously, rendering resistance breakdown statistically improbable [209,210]. This approach greatly enhances resistance durability by reducing the likelihood of pathogen adaptation and provides broad protection against diverse threats. Advanced gene stacking technologies enable the insertion of multiple receptor genes in one step, simplifying breeding and accelerating the development of resistant varieties. For example, transgenic wheat lines containing stacks of five to eight rust resistance genes demonstrated strong and lasting protection against multiple rust pathogens, illustrating the power of receptor gene pyramiding for sustainable crop protection [209]. Such stacked gene cassettes can be integrated through conventional breeding aided by molecular markers or through modern genetic engineering techniques like CRISPR, facilitating rapid deployment of complex resistance traits in wheat cultivars worldwide.

Manipulating wheat receptors for crop improvement has also greatly benefited from advances in genomics, genome-wide association studies (GWASs), and MAS. GWASs utilize diverse wheat populations to identify genetic loci, including receptor gene regions, associated with quantitative resistance and agronomic traits. High-resolution GWAS maps, coupled with quantitative trait loci (QTL) analyses, enabled precise detection of key loci for targeted traits, enhancing the efficiency of MAS to introgress favorable receptor alleles and pyramid multiple resistance genes [207]. Integrating receptor genes from wild relatives broadens genetic diversity, providing a crucial resource for sustainable wheat improvement programs focused on stress resilience and productivity. This integrated approach, combining molecular biology, genomics, and traditional breeding, accelerates the exploitation of PMRs to develop superior crop varieties. The feasibility of synthetic receptor engineering in wheat is advancing in parallel with the increasing availability of high-resolution structural and ligand-binding data for wheat RLKs. For example, structural modeling of receptors, such as TaFLS2 and TaLRR-RLK39, identified key amino acid residues essential for ligand perception. This knowledge enables the rational design of synthetic ectodomains with enhanced binding affinity for conserved pathogen-associated molecules, such as the chitin fragments prevalent in major wheat pathogens like *Fusarium* and *Zymoseptoria* spp. These engineered ectodomains can be fused to robust, well-characterized wheat kinase domains to generate fully orthologous synthetic receptors. This strategy creates tailored receptors for heightened immunity while circumventing the potential complications of cross-species domain integration [210].

## 8. Challenges and Limitations in Studying the Wheat PMRs

Studying wheat receptors is inherently challenging due to the hexaploid nature of wheat, which contains three related genomes (A, B, and D subgenomes), resulting in extensive gene redundancy and complexity. This genome complexity complicates the accurate identification and functional characterization of receptor genes, as researchers must distinguish between closely related paralogs and alleles scattered across the subgenomes. The large and repetitive wheat genome also poses technical difficulties in genomic analyses and bioinformatic annotation, requiring sophisticated approaches to resolve gene copies and sequences (Figure 7). While the total number of PMRs in wheat is still under investigation, many remain unidentified due to gaps in current genome annotations. However, with the recent release of the high-quality reference genome from the IWGSC, coupled with advances in bioinformatics and AI-based approaches, we expect that a more comprehensive annotation will be possible in the near future. In addition to genomic intricacy, the diverse receptor families in wheat add another layer of complexity. PMRs such as LRR-RLKs, lectin receptor-like kinases, and WIRKs exhibit a wide range of ligand specificities and dimerization behaviors, complicating the identification of their natural ligands and downstream signaling partners. Experimental methods to isolate and functionally analyze these membrane proteins face significant challenges due to their hydrophobic nature and low abundance. Functional redundancy among receptor paralogs further obscures phenotypic analysis, often masking the impact of single-gene mutations and necessitating advanced multiplex gene editing or allele stacking strategies for effective functional dissection.

Moreover, wheat receptor expression is highly dynamic, varying with tissue type, developmental stage, and environmental conditions such as biotic and abiotic stresses. This context-dependent expression necessitates integrative research approaches that combine genomics, transcriptomics, proteomics, and phosphoproteomics to fully unravel receptor functions within complex signaling networks. Receptors act within intricate signaling cascades characterized by cross-talk and feedback loops, making it difficult to isolate the contribution of individual receptors. Addressing these challenges requires multi-dimensional datasets and advanced computational modeling to understand the wheat receptors’ role in growth, development, and stress response. Such comprehensive analyses are essential for harnessing wheat receptor biology to improve crop performance and resilience.

## 9. Future Directions

Future research must first address the fundamental complexities of wheat PMRs by integrating multi-omics with precision genome engineering. A key strategy will be to employ CRISPR/Cas-mediated multiplex editing to dissect genetic redundancy across the A, B, and D subgenomes and to identify critical alleles for disease resistance and stress tolerance. Concurrently, long-read sequencing and pan-genome analyses will resolve complex paralogous clusters and uncover valuable alleles from diverse germplasm. At the molecular level, structural studies using cryo-electron microscopy and ligand profiling are vital to elucidate the mechanics of receptor-ligand interactions. Furthermore, phosphoproteomic and interactome mapping under stress will define the downstream signaling cascades, identifying new targets for breeding.

Building on these discoveries, the second phase involves integrating systems biology with translational breeding to engineer resilient wheat varieties. Machine learning models can predict optimal, synergistic combinations of receptor genes to design durable, broad-spectrum resistance. Incorporating this receptor data into genomic selection and GWAS pipelines will accelerate the introgression of elite alleles from wild relatives, enhancing genetic diversity (Figure 7). Synthetic biology approaches, such as engineering stress-inducible promoter systems, could optimize resource allocation. Ultimately, the potential of these advanced lines must be proven through large-scale field validation under diverse environmental and pathogen pressures, ensuring that laboratory breakthroughs translate into stable, high-performing cultivars equipped to meet future climate and disease challenges.

## 10. Conclusions

Wheat PMRs form a highly diverse sensory system essential for environmental perception. This review details their structural and functional diversity, amplified by the hexaploid genome, and their central roles in immunity. Despite research challenges posed by wheat’s complex hexaploid genome, these receptors show remarkable diversity, with families ranging from a few to over 3424 members. They are crucial for immunity, detecting biotic threats like pathogens and herbivores to initiate defense responses. Receptor cross-talk between growth and defense pathways ensures balanced responses, though it complicates targeted manipulation. Emerging tools in multi-omics, bioinformatics, and genome editing accelerate receptor discovery and validation, enabling strategies such as marker-assisted selection, gene stacking, and genetic engineering to enhance crop resilience. However, several key limitations must be acknowledged to accurately frame future efforts. Addressing these gaps requires a targeted roadmap: (i) systematic pan-genomic mapping of receptor alleles; (ii) deployment of multiplex and base-editing CRISPR platforms; (iii) integration of structural modeling to guide synthetic PMR design; (iv) strategic pyramiding of complementary receptors for durable resistance; and (v) coordinated phenotyping and translational pipelines to accelerate development. Collectively, these directions establish a clear technical path toward next-generation wheat improvement built upon a mechanistic understanding of PMR diversity, signaling, and engineering potential.

## Figures and Tables

**Table 1:** Techniques used for studying wheat receptors.

	Method	Principle	Example
Genomic and molecular techniques	Genome Sequencing	Sequencing the wheat genome to identify receptor-like protein genes via conserved domains.	LysM-RLKs LRR-RLKs CR-RLKs
	PCR-Based Cloning	Selective PCR amplification of receptor genes from genomic DNA or cDNA using gene-specific primers.	CRK
	RACE	A PCR-based technique designed to obtain the cDNA sequence by amplifying the 5′ and/or 3′ ends of the transcript.	CRK
	TILLING	Reverse genetics screening of mutagenized DNA to detect point mutations in target receptor genes.	Stb6
	CRISPR-Cas9	CRISPR-Cas9 system using gRNA to induce targeted knockout mutations in receptor genes.	CERK1 RPK1
Transcriptomic approaches	Microarray Analysis	cDNA microarray hybridization to measure relative abundance of predefined transcripts.	RLK
	RNA-seq	High-throughput cDNA sequencing to profile the transcriptome and analyze gene expression.	CRK
	qRT-PCR	Fluorescence-based qPCR to quantify expression of specific genes and validate transcriptomic data.	CERK1 Pm3
	scRNA-seq	Sequences the transcriptome of individual cells, revealing cell-type-specific expression patterns.	RLK
Proteomic and protein interaction approaches	Y2H	Screens for binary protein interactions by reconstituting a transcription factor in yeast.	TaXa21
	Co-IP	Uses an antibody to purify a target protein and its bound partners from a cell lysate.	LecRLKs RLK
	MS Proteomics	Identifies and quantify proteins from digested peptides based on mass-to-charge ratio, often coupled with LC for separation.	CERK1 RLK WAK1
	BiFC	Two non-fluorescent fragments of a fluorescent protein are fused to putative interacting partners; interaction reconstitutes fluorescence	Pm55

**Table 2:** Wheat receptor-like kinases representing their ligands and functional roles.

RLKs	Primary Ligand	Key Wheat Examples & Function
CRKs	ROS, unknown PAMPs/DAMPs	TaCRKs; stress-responsive signaling.
SD-RLKs	Carbohydrates (e.g., chitin), unknown ligands	LRK10L; confers powdery mildew (Bgt) resistance.
LRR-RLKs	Diverse: PAMPs, effectors, hormones (e.g., BR)	Lr34 (broad-spectrum), TaTLRK-6D (FHB), Yr5/Yr7 (stripe rust).
WAKs	Pectin, cell wall damage	TaWAK6 responds to leaf rust; cell wall integrity sensors.
PERKs	Cell wall integrity/perturbation	Large family (~30–37 genes); highly stress-responsive.
CR4	Unknown peptide	Putative regulator of grain and epidermal development.
ML-LRR-RLKs	Possibly glycans	RLK-V from wild wheat; confers powdery mildew resistance.
LysM-RLKs/CERKs	Fungal chitin oligosaccharides	TaCEBiP & TaCERK1; cooperate for FHB and other fungal defenses.
LecRLKs	Fungal carbohydrates (chitin, β-glucans)	LecRK-V (powdery mildew), TaSRLK (stripe rust).
FLS2	Bacterial flagellin	Conserved bacterial PPR; basal immunity.
SERKs	Co-receptor for multiple RLKs	TaSERK1; mediates defense and development pathways.
BRI1	Brassinosteroids	Central growth regulator; target for dwarfing alleles.
PSKRs	Phytosulfokine peptide	Promotes cell proliferation; integrates growth & stress.
SRKs	Pathogen effectors? (neofunctionalized)	Tandem kinases partnering with NLRs for rust resistance.
WIRKs	Damage signals, PAMPs	TtdLRK10L-1; rapid defense activation post-wounding.
GLKs	Scaffold for ABA/ROS signals	Pseudokinase; regulating stomatal closure; drought tolerance.

## Data Availability

No new data were created or analyzed in this study. Data sharing is not applicable to this article.

## Abbreviations

The following abbreviations are used in this manuscript:

PMRs	Plasma Membrane Receptors
PPI	Protein–Protein Interaction
PTMs	Post-Translational Modifications
PRRs	Pattern Recognition Receptors
LysM	Lysin Motif
LysM-RLKs	LysM Receptor-Like Kinases
LRR	Leucine-Rich Repeat
RLKs	Receptor-Like Kinases
CRKs	Cysteine-rich Receptor-Like Protein Kinases
RACE	Rapid Amplification of cDNA Ends
TILLING	Targeting Induced Local Lesions in Genomes
RNA-seq	RNA Sequencing
qRT-PCR	Quantitative Real-Time PCR
scRNA-seq	Single-Cell RNA Sequencing
MS	Mass Spectrometry
LC-MS/MS	Liquid Chromatography–Tandem MS
WAK1	Wall-Associated Kinase 1
Y2H	Yeast Two-Hybrid
Co-IP/MS	Co-Immunoprecipitation Coupled with Mass Spectrometry
LecRLKs	Lectin Receptor-Like Protein Kinases
BiFC	Bimolecular Fluorescence Complementation
PAMPs	Pathogen-Associated Molecular Patterns
DAMPs	Damage-Associated Molecular Patterns
SD-RLKs	S-Domain Receptor-Like Kinases
LRK10L-RLK	L-Lectin Receptor-Like Kinase 10L
LRR-RLKs	Leucine-Rich Receptor-Like Protein Kinases
FHB	Fusarium Head Blight
ETI	Effector-Triggered Immunity
HR	Hypersensitive Response
WAKs	Wall-Associated Kinases
PERKs	Proline-rich/Extensin-Like Receptor Kinases
ML-LRR-RLKs	Malectin-Like Leucine-Rich Repeat Receptor-Like Kinases
ROS	Reactive Oxygen Species
MAPK	Mitogen-Activated Protein Kinase
PR	Pathogenesis-Related
FLS2-RLKs	Flagellin-Sensing 2 Receptor Kinases
CERKs	Chitin Elicitor Receptor Kinases
SERKs	Somatic Embryogenesis Receptor Kinases
ABA	Abscisic Acid
BRI1	Brassinosteroid-Insensitive 1
BR	Brassinosteroid
PSKRs	Phytosulfokine Receptors
PSK	Phytosulfokine hormone
GC	Guanylate Cyclase
cGMP	Cyclic Guanosine Monophosphate
CDPKs	Calcium-Dependent Protein Kinases
SRKs	S-locus Receptor Kinases
NLR	Nucleotide-binding Leucine-rich Repeat
WIRKs	Wound-Induced Receptor Kinases
GLKs	GHR1-Like Kinases
GHR1	Guard Cell Hydrogen Peroxidase-Resistant 1
GPCRs	G Protein-Coupled Receptors
WLRK	Wheat Leaf Rust Kinase
HAMPs	Herbivore-Associated Molecular Patterns
PTI	Pattern-Triggered Immunity
RLCKs	Receptor-Like Cytoplasmic Kinases
TaFLS2-like	Wheat Flagellin Sensing 2
BAK1	BRI1-Associated Kinase 1
TIR	Toll/Interleukin-1 Receptor
NB	Nucleotide-Binding
R	Resistance Genes
VOCs	Volatile Organic Compounds
AM	Arbuscular Mycorrhizal
RLPs	Receptor-Like Proteins
LBDs	Ligand-Binding Domains
BIN2	Brassinosteroid-insensitive 2
JA	Jasmonic Acid
EFR	EF-Tu Receptor
MAS	Marker-Assisted Selection
KASP	Kompetitive Allele Specific PCR
GWAS	Genome-Wide Association Study
QTL	Quantitative Trait Loci
CrRLK1L	*Catharanthus roseus* RLK1-like
PYL	Pyrabactin resistance 1-like
RPK1	Receptor-Like Protein Kinase 1
CRs	Cytoplasmic Receptors
ORs	Organelle Receptors
PKs	Protein Kinases
SAR	Systemic Acquired Resistance
